# Early detection and prevention

**DOI:** 10.1002/1878-0261.12459

**Published:** 2019-02-27

**Authors:** Joakim Dillner

**Affiliations:** ^1^ Center for Cervical Cancer Prevention Department of Pathology Karolinska University Laboratory and Karolinska Institutet Stockholm Sweden

**Keywords:** cancer, Cancer Prevention Europe, detection, prevention, screening

## Abstract

The rapidly increasing incidence and mortality of cancer calls for a focused effort to increase the effect of cancer‐prevention efforts. In the area of early detection, there are major differences in the preventive impact of implemented screening policies, even when solid, evidence‐based international recommendations are issued. Studies are needed to determine why evidence‐based interventions are not used and to investigate why effects are less than predicted by solid research on the subject. Currently, population‐based screening is recommended only for three forms of cancer (cervical, breast and colorectal cancer) but, given the increasing cancer burden, efforts are required to facilitate the discovery of new biomarkers for screening, as well as the identification of barriers to implementation of new cancer screening discoveries. The creation of a network of excellence in research on Cancer Prevention (Cancer Prevention Europe) is likely to significantly contribute to progress in these areas. In the present review, some possible strategies to ensure progress are discussed, with specific examples from the cervical cancer screening area.

**Abbreviations**
AGUS: atypical glandular cells of undetermined significance
GDPR: General Data Protection Regulation
HPV: human papillomavirus
IARC: International Agency for Research on Cancer
IT: information technology
Pap: Papanicolaou
RHP: randomized healthcare policies
RR: relative risk
WHO: World Health Organization

## 1 Introduction

The global incidence of new cancers in 2018 was estimated at 18 million cases, with a projected increase to 30 million new cases per year (*a* + 62% increase) by 2040 (https://gco.iarc.fr/). With the rapidly increasing costs for cancer treatment, the cancer epidemic poses a threat to global welfare advancement, calling for development of effective cancer prevention as a major societal challenge. Early detection has two major components: screening and education to recognize symptoms and promote an early diagnosis. The present review focuses on screening. Although screening is recognized as a major part of cancer‐preventive policies, there are only three cancer forms for which organized screening is recommended and, in many cases, such policies have not been implemented or have not achieved the expected health benefits (Basu *et al*., 2018).

The major challenges in the area of secondary cancer prevention can thus be structured as:

- Assessing whether there is sufficient evidence to recommend screening for a particular form of cancer.
- Assessing barriers that impede implementation of evidence‐based screening policies.
- Assessing reasons for lower than expected effects of actually implemented programs and how the effectiveness of real‐life programs can be optimized.
- Furthering a research infrastructure that allows for discovery of new biomarkers potentially useful for screening.

The major challenges in these different areas are briefly discussed, including some possible strategies to address them. Most of the examples are taken from our experience with cervical cancer screening, although the issues are generic and applicable to screening for any form of cancer.

## 2 Assessing evidence

In the overall assessment of the evidence to underpin screening, a format for structured assessment of all the evidence has been established for more than 20 years (Richardson *et al*., 1995). The model Population, Intervention, Control group, Outcome and Study design includes that a team of clinical experts should assess which clinical questions should be prioritized and also define how to answer them, by a Population, Intervention, Control group, Outcome and Study design (Table 1). An independent team of data abstractors extract data from the entire scientific literature and grade the evidence in defined steps of recommendations, with an associated grading of the state of the evidence. The process is overseen by an editorial board, ensuring that it is correctly performed (Minozzi *et al*., 2012). Somewhat surprisingly, it is not uncommon that different agencies performing the same systematic review (SR) on the same issue arrive at different conclusions. There are even examples of the same agency performing the same review twice, with opposite conclusions. By design, the process should be operator‐independent and reproducible but, currently, the evidence assessment is often performed both by international agencies, then repeated by national agencies and then also by regional agencies, resulting in a slow adaption of new policies. There is a tendency to primarily use evidence assessments performed by highly credible agencies or networks, such as the reviews performed by the World Health Organization (WHO)/International Agency for Research on Cancer (IARC) on behalf of the European Commission or the reviews by the Cochrane collaboration, although this is not in line with the idea that a reproducible process should be operator‐independent. Also, if discussions about whether to implement or not focus on credibility rather than on the evidence, it becomes difficult for the research community to focus on answering the most important research questions where additional evidence might be needed for implementation.

**Table 1:** The established system for assessing evidence (Minozzi *et al*., 2012).

P	Population	How would I define the population?
I	Intervention, prognostic factor, or exposure	Which main intervention am I considering?
C	Comparison group	Which comparison (control) group is adequate to compare the intervention with?
O	Outcome you would like to measure or achieve	What can I hope to accomplish, measure, improve, or affect?
S	Study design	What would be the appropriate study design(s)/methodologies?

Grading of recommendations and supporting evidence for the level of evidence:

(I) Consistent multiple randomised controlled trials (RCTs) of adequate sample size, or SRs of RCTs, taking into account heterogeneity.

(II) One RCT of adequate sample size, or one or more RCTs with small sample size.

(III) Prospective cohort studies or SRs of cohort studies; for diagnostic accuracy questions, cross‐sectional studies with verification by a reference standard.

(IV) Retrospective case–control studies or SRs of case–control studies, trend analyses.

(V) Case series; before/after studies without control group, cross‐sectional surveys.

(VI) Expert opinion. For the strength of the respective recommendation:

(a) intervention strongly recommended for all patients or targeted individuals;

(b) intervention recommended;

(c) intervention to be considered but with uncertainty about its impact;

(d) intervention not recommended; and

(e) intervention strongly not recommended.

## 3 Barriers that impede implementation of evidence‐based screening policies

Even if the evidence is reproducibly assessed as sufficient to recommend a screening policy that can contribute to controlling the cancer burden, it is very common that implementation does not happen. For example, based on the WHO/IARC reviews, the European Commission has recommended population‐based, organized screening programs for three forms of cancer (cervical, breast and colorectal), although an assessment of whether these policies had actually been implemented in Europe found varying degrees of implementation, even though some policies have been recommended for more than 50 years (Basu *et al*., 2018). Interestingly, new recommendations appeared to spread faster than old ones (Basu *et al*., 2018), implying that, in some instances, pre‐existing structures may be a barrier rather than an advantage for implementation.

Some conceivable bottlenecks for implementation are:

- Lack of community support: Lack of political support is also sometimes mentioned as a barrier, which is (at least in democracies) related to the community support. An interesting example of a strategy is the global cervical cancer elimination initiative, a call to action from the Director of the WHO. The strategy (for each country) starts with building the case for the initiative; for example, in terms of health benefits, impact on the economy and welfare of society. This is then coupled with a practical inventory of what is needed to succeed and an associated communications strategy. Once strong community support is achieved, remaining bottlenecks are unlikely to remain.
- Money: Lack of resources is commonly cited as a problem, which is unfortunately seen even for cancer control strategies that are cost‐saving. Cancer treatments and palliative care are expensive and cervical cancer screening is an example of a policy that is cost‐saving to society. A problem is that local health economy studies commonly use an existing policy as the base case for calculating cost‐effectiveness and do not consider alternative strategies that would be less wasteful (more cost‐efficient). Examples include over‐use of screening (disorganized rather than organized screening and screening in shorter intervals than necessary), use of overly expensive tests and information technology (IT) systems (e.g. studies ignoring effects of market shaping such as large‐scale tenders), sampling strategies (e.g. physician‐taken samples rather than nurse‐taken samples or self‐collected samples) or treatments. More modelling and health economics research focusing on inexpensive ways to achieve the desired health effects would be important.
- No access to data: Population‐based screening has as the first basic step to identify the target population that should be screened. In countries with population‐based screening, lists with identities of subjects residing in the country (that should be targeted for population‐based screening) are usually provided by the Tax Office. All countries have censuses and levy taxes, although screening programs do not always have access to these population lists. It does not seem congruent to decide to launch a screening program without facilitating the most basic prerequisites, such as access to the lists of who should be screened.

A similar problem that is sometimes raised is a lack of access to data from cancer registries and/or screening registries, such that the program cannot be monitored and evaluated. If a screening registry does not exist, it is simple to build one (Elfstrom *et al*., 2016; Hortlund *et al*., 2018). A survey found that most European countries did have a screening registry (Elfstrom *et al*., 2015) but, sometimes, it was not used for the follow‐up and optimization of the program (Elfstrom *et al*., 2015), suggesting that more research is needed on the most effective uses of a screening registry to achieve an optimally cost‐effective program by incremental, evidence‐based improvements.

- IT: Problems with launching and maintaining an adequate IT structure is another possible bottleneck. Many successful screening programs and screening registries were started in the 1950s, at a time when IT technology was not very advanced. This implies that the required IT support is actually rather basic. There is now widespread IT literacy with good coding knowledge in most societies worldwide, which should facilitate greater access to relevant systems that are adaptable to changing guidelines and program needs. The Open Science concept (which includes that all software used should be freely available for others to use and adapt) has unfortunately not yet been widely adopted in the screening setting. Major funding agencies and journals today insist on Open Science, and also that the software used must be Open Source. If the software used for screening was indeed made openly available in public repositories, its adaptation to other screening projects in other settings would be simple. Most likely, this would result in optimal screening practices being developed and spreading much faster.

## 4 Achieving full effectiveness: follow‐up and incremental optimization of programs

Although the process for how to collect follow‐up data and use it for a continuous optimization of the screening program is clearly described in the literature, it is not always widely understood. In 1968, the *Principles and Practices of Screening for Disease* was published by Wilson and Jungner (1968), including 10 classic criteria for evaluating screening. With limited modifications, these criteria are still used to evaluate screening strategies to this day. However, there are many examples of where the actual effect of an implemented screening program is much less than predicted from studies in the research setting, suggesting that research is needed on what the barriers to effective implementation are and how they can be overcome to achieve optimization of programs.

A prime example of the problem is screening for cervical cancer. The Papanicolaou (Pap) smear was described in the 1930s and, although there is established evidence that women who regularly attend a Pap smear screening program reduce their risk for cervical cancer by approximately 90%, no country has actually achieved such a high impact. By contrast, the incidence of this cancer in Europe varies by more than 10‐fold, with strong variability in impact also among countries who have implemented this screening.

An important conceptual advance came from a group of distinguished scientists in the area who jointly proposed that regular audit was an ethical requirement of screening (Sasieni and Cuzick, 2001). Screening programs have both adverse effects (e.g. turning healthy individuals into patients) and beneficial impacts on health that are not consistently fully realized in real‐life. The reasoning thus goes that the launching of a screening program without auditing what the adverse effects are and whether the health gains are actually realized is not ethical. Auditing has been a part of the European Union recommendations for cervical screening for many years, although a Europe‐wide survey found that only 12 countries were actually performing audits, that only six of these countries were doing audits with a comparison group and that, even when audits were performed, there was commonly no budget for them, implying limited sustainability (Elfstrom *et al*., 2015).

The basic idea of the audit is simple. Among all cases of disease occurring in the population, which components of the preventive services have actually reached the individuals who nevertheless developed the disease? When comparing with the population that did not develop disease, were they reached by preventive services to the same extent and are there any differences that might explain why the cancer did develop even though the population was targeted for screening? For each possible improvement of the screening program, the effect can be calculated as S*(1 – 1/RR), where *S* is the proportion of the cancer cases affected and RR is the relative risk for cancer. As a specific example, if 9% of cancer patients have a history of a screen‐detected abnormality that was never treated and the associated RR for cancer is 20, then 9% × 0.95 = 8.5% of cancers can be prevented if all women with abnormal screens are indeed treated.

Interestingly, the occurrence of abnormal screens that are not followed up is an important cause of cancer in Sweden, although it appears to be absent in neighboring countries. There are also some counties in Sweden where 100% of women with abnormal screens are indeed followed up (www.nkcx.se) (Hortlund *et al*., 2018).

Incremental optimization refers to a continuous process of improving a screening program to obtain better effectiveness. When a Ministry of Health or equivalent launches a program and the framework of its design, the program is usually also tasked with ensuring that the effectiveness is as high as possible and that costs and adverse effects are contained. The exact tasks involved will differ depending on the maturity of the program, associated expertise and opportunities. Information from an audit may serve as a basis for incremental optimization efforts. For example, the audit of the cervical screening program in Sweden found that there were four main areas where improvements were likely to result in health gains.

- Improving long‐term attendance. Only 2% of the target population did not take a screening test over an interval of 8 years. At the same time, this small proportion of the population generated 38% of the cervical cancer cases. Sending self‐sampling kits to the long‐term non‐attenders resulted in additional attendance and very high positive predictive value (38%) for high‐grade intraepithelial neoplasia (CIN2+) among screen‐positives (report available at: hpvcenter.se)
- A high proportion of cervical cancer deaths was found to occur after the stop of the screening program at 60 years of age. A nationwide registry linkage found that these deaths occurred among women who had either not been screened between age 50 and 60 years or had received an abnormal screening result (Wang *et al*., 2017). Consequently, the program was changed to extend the invitations up to 70 years of age, requiring that women had a normal smear taken at 64 years of age or later (Socialstyrelsen (The National board for Health and Welfare), 2015).
- Improving the protection against cervical adenocarcinoma. Although the program was found to have very strong protection against cervical squamous cell carcinoma, the protection against cervical adenocarcinoma was more modest. A linkage of the screening registry and the cancer registry found that the uncommon cytologic finding glandular atypia (AGUS) had higher risks for subsequent cervical cancer than even the high‐grade squamous epithelial lesions (CIN2+), that only about half of these lesions had resulted in a treatment and that, in contrast to treatment of CIN2+, the cancer risk declined only marginally after treatment (Wang *et al*., 2016). The conclusion was that treatment must be improved and that follow‐up to ensure that women with AGUS are treated is warranted. Indeed, the positive predictive value for diagnosis of a CIN2+ lesion among women referred for human papillomavirus (HPV)‐positive AGUS was as high as 60% (Norman *et al*., 2017). In addition, because the proportion of smears diagnosed with AGUS varied 40‐fold between laboratories (www.nkcx.se), quality assurance of the diagnosis of AGUS was warranted.
- Improving the sensitivity of the screening test. Joint European randomized trials found that, over 8 years, the protection against cervical cancer was approximately twice as high with the HPV‐test as with the cytological screening (Ronco *et al*., 2014). Subsequent to 2008, European guidelines have recommended HPV testing as an alternative to cytology (Directorate‐General for Health and Food Safety, 2008) provided that the new test was introduced in a carefully controlled and evaluable manner (randomized healthcare policies, RHP) (Hakama *et al*., 2012). However, only a few countries (e.g. Finland and Sweden) implemented RHPs. In 2014/2015, both the WHO and the European Union (Directorate‐General for Health and Food Safety, 2015; World Health Organization, 2014) recommended the HPV test for routine use and, subsequently, a large number of countries have implemented it. It is noteworthy that changing the screening test was a considerably more lengthy process than the various incremental optimizations that were identified by the screening registry. Without the RHP to test the effectiveness in real‐life conditions, it is likely that the implementation process would have taken even more time.

For example, a new national management guideline was decided in 2017, which included new algorithms for the treatment and follow‐up of AGUS (see above). Also, a registry linkage study found that treatment of women with low‐grade dysplasia had no cancer‐preventive effect for women below 28 years of age (Sundstrom *et al*., 2017) and the policy to refer young women with low‐grade dysplasia to colposcopy and biopsy was therefore abolished in a national management guideline issued just 6 months later. By contrast, HPV‐based screening is still only used in seven out of 21 counties in Sweden, 3.5 years after a governmental decision to implement it. Again, the most commonly cited reasons for non‐implementation are money and IT, although the counties in Sweden that did implement it report that HPV was cost‐neutral (i.e. no additional money was required) and that no IT‐modifications were required. Why some evidence‐based improvements are rapidly implemented, but others take more time is not entirely clear.

## 5 Furthering a research infrastructure for biomarker discovery and validation

**Figure 1:**
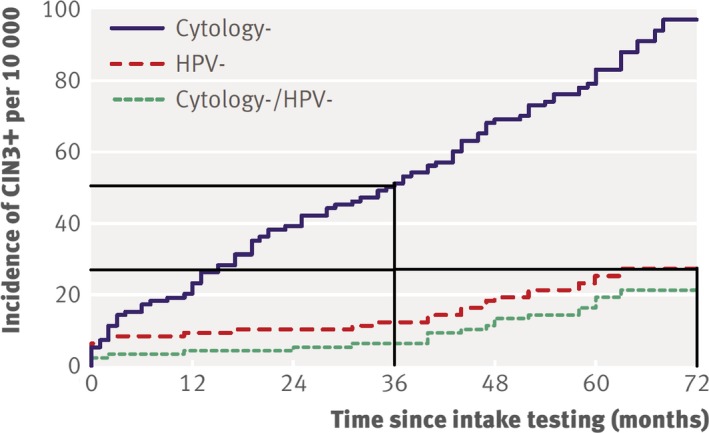
Graph of the main findings of some 20 years of research on HPV testing for cervical screening, adapted from Dillner *et al*. (2008)). (a) The HPV test protects against cancer *in situ* for more than 6 years, whereas cytology only protects for 3 years. (b) No additional benefit of cytology among HPV‐negative women. The *y*‐axis displays the incidence of cervical cancer *in situ* or worse (CIN3+) in a joint European cohort assembled from five European Union countries. Cyt– indicates women who had negative cytology at baseline; hpv– indicates women who were HPV‐negative at baseline; cyt–/hpv– indicates women who were negative for both tests at baseline. As can be seen, the CIN3+ risk after 3 years for a cytology‐negative woman is about the same as the CIN3+ risk for an HPV‐negative woman after 6 years. This risk is very similar to the risk of women negative in both tests.

It is important to consider what could be done to promote effective research into finding new biomarkers suitable for screening and how the evaluation and implementation process could be made faster and simpler without compromising quality. The evaluation and implementation of HPV testing as a biomarker for cervical screening serves well to illustrate this process. In 1988, the first HPV test was granted Food and Drug Administration of the USA approval. Thirty years later, the evaluation and implementation process is still not complete. First, there was a phase where the performance of the HPV test in the screening setting was approximated using studies in archival Pap smears (Wallin *et al*., 1999). These studies had an important role in motivating funding of large randomized controlled trials, although they could not in themselves be used for decision‐making because the results on archival smears and on freshly collected samples were decidedly different. Cohort studies and randomized intervention trials based on HPV tests in freshly collected samples followed (Bulkmans *et al*., 2004; Giorgi‐Rossi *et al*., 2007; Kitchener *et al*., 2006; Naucler *et al*., 2007; Schlecht *et al*., 2001). Pooling of results from trials with cancer *in situ* as endpoint (Ronco *et al*., 2014) finally resulted in the demonstration of an improved effect on invasive cancer. The most important results of these studies are two‐fold: (a) The protective effect of testing negative for HPV lasts approximately twice as long as testing negative in a Pap smear. (b) The additional protective effect of testing all women with both HPV and Pap is very small compared to the protective effect of HPV testing alone. This is well explained by reference to Fig. 1, which displays joint data from European trials with 6 years of follow‐up. Because there were several years of planning, fund‐raising etc., before starting and, because there was also time required to gather and analyse the data, Fig. 1 is actually representative of approximately 10 years of work.

Our group has proposed that collection of samples from large cohorts, where the samples are stored in a manner that will mimic freshly collected samples and where the cohort is followed up with systematic registry linkages, will enable obtaining similar data but without having to wait for the outcomes. The example mentioned above, where extended waiting for outcomes to develop was required, resulted in a delay by a decade for women to benefit from HPV‐based screening by a decade. The proposed resource not only will enable the discovery and validation of new biomarkers, but also will be useful for studies on equivalence of new variants of previous tests. An example is a recent validation study where an HPV test based on detection of HPV mRNA was found to have an equivalent performance to tests based on the detection of HPV DNA over a 7‐year follow‐up (Forslund *et al*., 2019). The study was based on a large biobank collected more than 7 years ago that had been followed up with registry linkages – and thus did not have to wait prospectively for 7 years to obtain long‐term follow‐up data. Another example is a recent validation of a new version of the Cobas 4800 HPV test, the Cobas 6800 HPV test, which was found to have an identical performance as the older version of the test when using a large biobank of cervical smears followed up for future development of CIN3+ (cancer *in situ* or worse) in histopathology as the basis for the comparison.

The research infrastructure for development, evaluation and incremental optimization of cancer screening is heavily dependent on the ability to perform population‐based registry linkages. For several years, the cancer prevention community has debated whether the 2018 European Union General Data Protection Regulation (GDPR) will result in impaired opportunities for effective cancer prevention because the basic principle of GDPR is either individual consent (which is not possible for population‐based linkages) or anonymization (which does not allow any linkages). However, the final version of the GDPR contains an exception for research (van Veen, 2018) and with good governance and strong community support, continued development of effective cancer screening is likely to be possible also in the future.

Today, both European and national guidelines on cervical screening recommend systematic biobanking, both as a resource for quality assurance and for research and development. The practicalities of the biobanking process have been described and there was also a recent comparison of the Swedish and Scottish cervical screening biobanks (Alcañiz Boada *et al*., in press).

For other cancer forms, longitudinally followed cohorts with blood samples may be particularly useful. There are indications that even cervical smear biobanks may be useful for the discovery of biomarkers for screening for other cancer forms because epigenetic modifications of DNA occur early in life, affect the risk for multiple cancer forms and can be measured in the DNA of cervical cells (Widschwendter *et al*., 2018). The concept to systematically link biobanks with cancer registries aiming to establish a study base with blood samples that can be used for longitudinal research on screening has been known for decades (Pukkala *et al*., 2007) and there are very large cohorts with blood samples established for cancer research broadly, such as the Janus cohort in Norway (Langseth *et al*., 2017) and the Finnish Maternity Cohort (Lehtinen *et al*., 2017), or more specifically for breast cancer screening, such as the Karma cohort in Sweden (Gabrielson *et al*., 2017). The latter allows for easy combination of biomarker data with detailed results from mammographic screening. There are many types of biomarkers that can be detected in blood samples (Pukkala *et al*., 2007). Well known examples include tumor antigens and circulating tumor DNA. More recent examples include cell‐free DNA/exosomes, microRNA and metagenomcis.

One additional advantage of population biobank‐based evaluation of biomarkers for screening should be highlighted. A large proportion of the literature on biomarkers today is based on samples collected particularly for validation of a particular biomarker. This results in it not being entirely clear which underlying population the samples refer to and, most importantly, it cannot be inferred how much a new biomarker adds to the predictive value of other biomarkers that are tested using other samples. By contrast, population‐based biobanks constitute an open access resource that can be requested by any researcher, anywhere in the world. Because it is good practice to deposit results of different studies at the biobank, an increasingly used biobank will accumulate the results of different biomarker tests from the same cases of disease that have occurred in the cohort, enabling comparison of whether a new biomarker adds anything to previously analysed biomarkers for the prediction of disease in a well‐defined population‐based cohort.

Apart from results obtained from biomarker analyses, additional data such as from previous screening results or provided by the patient himself/herself may further increase the predictive values. This is the basis of the popular concept of ‘risk‐stratified screening’ or ‘precision prevention’. It is vital that the public health orientation of cancer prevention is not lost by focusing on ever smaller target populations. Risk‐stratifications must encompass the entire population and not merely focus on identifying small high risk groups for screening. A recent example is the use of screening histories for risk stratification in cervical screening. Simply using the screening registry to classify previous screens as normal, absent or degrees of abnormal readily separated the population into strata with 10‐fold differences in cervical cancer risk (Baltzer *et al*., 2017). It seems wasteful to issue 3‐yearly screenings for everyone, when data that could guide the intensity for issuing of screening invitations based on risk is already on file. Clearly organized and population‐based screening programs with an accompanying screening registry and a population‐based biobank of the residual screening samples comprise one possible way of improving our ability to meet the societal challenge of better screening for better cancer prevention.

## Conflict of interest

Dr J. Dillner reports that his employer has contracts with Merck, Roche and Genomica.
